# An Adaptive Hybrid Algorithm Based on Particle Swarm Optimization and Differential Evolution for Global Optimization

**DOI:** 10.1155/2014/215472

**Published:** 2014-02-09

**Authors:** Xiaobing Yu, Jie Cao, Haiyan Shan, Li Zhu, Jun Guo

**Affiliations:** ^1^China Institute of Manufacturing Development, Nanjing University of Information Science & Technology, Nanjing 210044, China; ^2^Collaborative Innovation Center on Forecast and Evaluation of Meteorological Disasters, Nanjing University of Information Science & Technology, Nanjing 210044, China; ^3^School of Mechanic and Electronic Engineering, Wuhan University of Technology, Wuhan 430070, China

## Abstract

Particle swarm optimization (PSO) and differential evolution (DE) are both efficient and powerful population-based stochastic search techniques for solving optimization problems, which have been widely applied in many scientific and engineering fields. Unfortunately, both of them can easily fly into local optima and lack the ability of jumping out of local optima. A novel adaptive hybrid algorithm based on PSO and DE (HPSO-DE) is formulated by developing a balanced parameter between PSO and DE. Adaptive mutation is carried out on current population when the population clusters around local optima. The HPSO-DE enjoys the advantages of PSO and DE and maintains diversity of the population. Compared with PSO, DE, and their variants, the performance of HPSO-DE is competitive. The balanced parameter sensitivity is discussed in detail.

## 1. Introduction

Evolutionary algorithms (EAs), inspired by the natural evolution of species, have been successfully applied to solve numerous optimization problems in diverse fields [1]. Particle swarm optimization (PSO) and differential evolution (DE) are two stochastic, population-based optimization EAs [2].

PSO was introduced by Kennedy and Eberhart in 1995 [3, 4]. PSO uses a simple mechanism that mimics swarm behavior in birds flocking and fish schooling to guide the particles to search for globally optimal solutions. As PSO is easy to implement, it has rapidly progressed in recent years with many successful applications in solving real-world optimization problems. During the last decade, PSO algorithm has been paid much attention to in various areas [5–19] due to its effectiveness in handling optimization problems. Unfortunately, the PSO algorithm suffers from the premature convergence problem which does exist in complex optimization issues. In [17–19], some methods for tuning parameters including inertia weights and acceleration coefficients for PSO have been proposed to enhance PSO's search performance. A comprehensive learning PSO (CLPSO) algorithm was proposed in [14], which shows its superiority in dealing with multimodal functions.

DE is a simple yet powerful EA for global optimization introduced by Storn and Price [20]. The DE algorithm has gradually become more popular and has been used in many practical cases, mainly because it has demonstrated good convergence properties and is principally easy to understand [21]. DE has been successfully applied in diverse fields of engineering [22–26]. The performance of the conventional DE algorithm highly depends on the chosen trial vector generation strategy and associated parameter values used. Inappropriate choice of strategies and parameters may lead to premature convergence, which have been extensively demonstrated in [27]. In the past decade, DE researchers have suggested many empirical guidelines for choosing trial vector generation strategies and their associated control parameter settings [1, 28–31].

Although DE and PSO have been successfully applied to a wide range of problems including test and real life problems, both have certain shortcomings associated with them which sometimes deteriorate the performance of algorithms. The major problem is the lack of diversity resulting in a suboptimal solution or a slow convergence rate [32]. In order to improve the performance of these algorithms, one of the class of modified algorithms consists of the hybridization of algorithms, where the two algorithms are combined together to form a new algorithm. DE is applied to each particle for a finite number of iterations to determine the best particle which is then included into the population [33]. DE is applied to the best particle obtained by PSO [34]. A hybrid version of PSO and DE is proposed which is named Barebones DE [35]. The evolution candidate solution is generated either by DE or by PSO according to some fixed probability distribution [36]. A hybrid metaheuristic is designed so as to preserve the strengths of both algorithms [32].

However, it is worth mentioning that, in almost all the hybrid works mentioned above, the convergence rate is not fast enough or the global search performance is not satisfactory. To achieve the goal, a novel adaptive hybrid algorithm based on PSO and DE (HPSO-DE) is formulated by developing a balanced parameter between PSO and DE. Adaptive mutation is carried out to current population when the population clusters around local optima. The HPSO-DE enjoys the advantages of PSO and DE and maintains diversity of the population. With the efforts, the HPSO-DE has the ability to jump out of the local optima.

## 2. PSO and DE

### 2.1. PSO

In the standard PSO, a swarm consists of *m* individuals (called particles) that fly around in an *n-*dimensional search space. The position of the *i*th particle at the *t*th iteration is used to evaluate the particle and represent the candidate solution for the optimization problem. It can be represented as *X*_*i*_^*t*^ = [*x*_*i*1_^*t*^, *x*_*i*2_^*t*^,…, *x*_*in*_^*t*^], where *x*_*ij*_^*t*^ is the position value of the *i*th particle with respect to the *j*th dimension (*j* = 1,2,…, *n*). During the search process, the position of a particle is guided by two factors: the best position visited by itself (*p*_best_) denoted by *P*_*i*_^*t*^ = [*p*_*i*1_^*t*^, *p*_*i*2_^*t*^,…, *p*_*in*_^*t*^] and the position of the best particle found so far in the swarm (*g*_best_) denoted by *G* ^*t*^ = [*g*_1_^*t*^, *g*_2_^*t*^,…, *g*_*n*_^*t*^]. The new velocity (denoted by *V*_*i*_^*t*^ = [*v*_*i*1_^*t*^, *v*_*i*2_^*t*^,…, *v*_*in*_^*t*^]) and position of particle *i* at the next iteration are calculated according to

(1) $$\begin{array}{c} v_{ij}^{t+1}=w\times v_{ij}^{t}+c_{1}\times r_{1}\times \left(p_{ij}^{t}-x_{ij}^{t}\right)+c_{2}\times r_{2}\times \left(g_{j}^{t}-x_{ij}^{t}\right), \end{array}$$

(2) $$\begin{array}{c} x_{ij}^{t+1}=x_{ij}^{t}+v_{ij}^{t+1}, \end{array}$$

where *w* is the inertia weight, *c*_1_, and *c*_2_ are, respectively, the cognitive and social learning parameters, and *r*_1_, and *r*_2_ are random numbers between (0, 1). Based on the above equations, the particle can fly through search space toward *p*_best_ and *g*_best_ in a navigated way [16, 17].

### 2.2. PSO Variants

#### 2.2.1. PSO-w

In the PSO algorithm, proper control of global exploration and local exploitation is an important issue. In general, the higher values of inertia weight *w* help in exploring the search space more thoroughly in the process and benefit the global search, while lower values help in the local search around the current search space. The major concern of this linear PSO is to avoid the premature convergence in the early period of the search and to enhance convergence to the global optimum solution during the latter period of the search. The concept of linearly decreasing inertia weight was introduced in [17] and is given by

(3) $$\begin{array}{c} w=w_{\max}-\left(w_{\max}-w_{\min}\right)\left(\frac{\text{iter}}{{\text{iter}}_{\max}}\right), \end{array}$$

where iter is the current iteration number and iter_max⁡_ is the maximum number of iteration. Usually the value of *w* is between 0.9 and 0.4. Therefore, the particle is to use lager inertia weight during the initial exploration and gradually reduce its value as the search proceeds in further iterations. According to the research [37], the inertia weight is adjusted by (4). The nonlinear descending can achieve faster convergence speed than that with linear inertia weight:

(4) $$\begin{array}{c} w=\left(w_{1}-w_{2}\right)\times \frac{{\left(\text{iter}-{\text{iter}}_{\max}\right)}^{2}}{{\left({\text{iter}}_{\max}\right)}^{2}}+w_{2}, \end{array}$$

where *w*_1_ and *w*_2_ are the initial and final inertia weight.

#### 2.2.2. PSO-TVAC

Although linear PSO can locate satisfactory solution at a markedly fast speed, its ability to fine-tune the optimum solution is limited, mainly due to the lack of diversity at the latter stage of evolution process. In population-based optimization methods, the guideline is to encourage the individuals to roam through the entire search space during the early period of the search, without clustering around local optima. During the later period, convergence towards the global optima is encouraged [3]. With this view, a novel strategy in which time-varying acceleration coefficients are employed by changing the acceleration coefficients with time is proposed [16, 38]. With a large cognitive component and small social component at the beginning, particles are allowed to move around the search space, instead of moving toward the population best. On the other hand, a small cognitive component and a large social component allow the particles to converge to the global optima in the latter part of the optimization. This approach is referred to as PSO-TVAC. This modification can be mathematically represented as follows:

(5) $$\begin{array}{c} c_{1}=\left(c_{1f}-c_{1i}\right)\left(\frac{\text{iter}}{{\text{iter}}_{\max}}\right)+c_{1i},\\ c_{2}=\left(c_{2f}-c_{2i}\right)\left(\frac{\text{iter}}{{\text{iter}}_{\max}}\right)+c_{2i}, \end{array}$$

where *c*_1*i*_, *c*_1*f*_, *c*_2*i*_, and *c*_2*f*_ are initial and final values of cognitive and social acceleration factors, respectively; usually *c*_1*i*_ = *c*_2*f*_ = 2.5 and *c*_1*f*_ = *c*_2*i*_ = 0.5 [38].

### 2.3. DE

DE is proposed by Storn and Price [20]. It is an effective, robust, and simple global optimization algorithm. According to frequently reported experimental studies, DE has shown better performance than many other EAs in terms of convergence speed and robustness over several benchmark functions and real-world problems [39].

In DE, there are three operators: mutation, crossover, and selection. Initially, a population is generated randomly with uniform distribution; then the mutation, crossover, and selection operators are applied to generate a new population. Trial vector generation is a crucial step in DE process. The two operators mutation and crossover are used to generate the trial vectors. The selection operator is used to select the best trial vector for the next generation. The initialization and DE operators are explained briefly as follows [40].

DE starts with a population of NP  *D*-dimensional candidate solutions which may be represented as *X*_*i*,*G*_ (*i* = 1,2,…, NP) = {*x*_*i*,*G*_^1^, *x*_*i*,*G*_^2^,…, *x*_*i*,*G*_^*D*^}, where index *i* denotes the *i*th individual of the population, *G* denotes the generation to which the population belongs, and *D* is the dimension of the population.

The initial population should try to cover the entire search space as much as possible by uniformly randomizing individuals within the search space constrained by the minimum *X*_min⁡_ = {*x*_min⁡_^1^, *x*_min⁡_^2^,…, *x*_min⁡_^*D*^} and maximum *X*_max⁡_ = {*x*_max⁡_^1^, *x*_max⁡_^2^,…, *x*_max⁡_^*D*^} bounds. Thus, the initial population can be described as follows:

(6) $$\begin{array}{c} x_{i,0}=x_{\min}+\text{rand}\left(0,1\right)\times \left(x_{\max}-x_{\min}\right), \end{array}$$

where rand(0,1)∈[0,1] is a uniformly distributed random variable [40, 44].

*(1)  Mutation*. After initialization, DE utilizes the mutation operation to generate a trial vector *V*_*i*,*G*_ = {*v*_*i*,*G*_^1^, *v*_*i*,*G*_^2^,…, *v*_*i*,*G*_^*D*^} with respect to each individual in the current population. *V*_*i*,*G*_ can be produced by certain mutation strategy. For example, the five most frequently mutation strategies implemented in the DE are listed as follows [1]:

 DE/rand/1:
  (7) $$\begin{array}{c} V_{i,G}=X_{r_{1}^{i},G}+F\cdot \left(X_{r_{2}^{i},G}-X_{r_{3}^{i},G}\right), \end{array}$$
 DE/best/1:
  (8) $$\begin{array}{c} V_{i,G}=X_{\text{best},G}+F\cdot \left(X_{r_{1}^{i},G}-X_{r_{2}^{i},G}\right), \end{array}$$
 DE/rand-to-best/1:
  (9) Vi,G=Xi,G+F·(Xbest,G−Xi,G)+F·(Xr1i,G−Xr2i,G),
 DE/best/2:
  (10) Vi,G=Xbest,G+F·(Xr1i,G−Xr2i,G)+F·(Xr3i,G−Xr4i,G),
 DE/rand/2:
  (11) $$\begin{array}{c} V_{i,G}=X_{r_{1}^{i},G}+F\cdot \left(X_{r_{2}^{i},G}-X_{r_{3}^{i},G}\right)+F\cdot \left(X_{r_{4}^{i},G}-X_{r_{5}^{i},G}\right). \end{array}$$

The indices *r*_1_^*i*^, *r*_2_^*i*^, *r*_3_^*i*^, *r*_4_^*i*^, and *r*_5_^*i*^ are mutually exclusive integers randomly generated within the range [0,1], which are also different from the index *i* [1]. *F* is the mutation scale factor which is used in controlling the amplification of the differential variation [40].

*(2)  Crossover*. The crossover operation is introduced to increase the diversity of the target vectors. After the mutation phase, the crossover operation is applied to *V*_*i*,*G*_ = {*v*_*i*,*G*_^1^, *v*_*i*,*G*_^2^,…, *v*_*i*,*G*_^*D*^} and *X*_*i*,*G*_ = {*X*_*i*,*G*_^1^, *X*_*i*,*G*_^2^,…, *X*_*i*,*G*_^*D*^} to generate a trial vector *U*_*i*,*G*_ = {*u*_*i*,*G*_^1^, *u*_*i*,*G*_^2^,…, *u*_*i*,*G*_^*D*^} as follows:

(12) $$\begin{array}{c} u_{i,G}^{j}=\{\begin{array}{cc} v_{i,G}^{j} & \text{if}\;{\text{rand}}_{j}\left[0,1\right)\leq \text{CR}\;\text{or}\;\;\left(j=j_{\text{rand}}\right),\\ x_{i,G}^{j} & \text{others}. \end{array} \end{array}$$

CR ∈ [0,1] is the crossover constant, which has to be determined by the user. *j*_rand_ ∈ [1, *D*] is a randomly chosen index which ensures that the trial vector *U*_*i*,*G*_ will differ from *X*_*i*,*G*_ by at least one parameter.

*(3)  Selection*. If the generated trial vector *u*_*i*,*G*_^*j*^ exceeds the corresponding upper and lower bounds, we randomly and uniformly reinitialize it within the search range. Then the fitness values of all trial vectors are evaluated [45].

After that, a greedy selection scheme is employed:

(13) $$\begin{array}{c} x_{i,G+1}=\{\begin{array}{cc} u_{i,G} & \text{if}\;\;f\left(u_{i,G}\right)\leq f\left(x_{i,G}\right),\\ x_{i,G} & \text{otherwise}. \end{array} \end{array}$$

If the trial vector *u*_*i*,*G*_ yields a better cost function value than *x*_*i*,*G*_, the *u*_*i*,*G*_ will replace *x*_*i*,*G*_ and enter the population of the next generation; otherwise, the old value *x*_*i*,*G*_ is retained.

### 2.4. DE Variants

In order to improve the performance of DE, some adaptive DE variants are proposed. jDE was proposed based on the self-adaptation of the scale factor *F* and the crossover rate CR [29]. The jDE is represented by a *D*-dimensional vector *X*_*i*,*G*_ (*i* = 1,2,…, NP). New control parameters or factors *F*_*i*,*G*+1_ and CR_*i*,*G*+1_ are calculated as

(14) $$\begin{array}{c} F_{i,G+1}=\{\begin{array}{cc} F_{l}+{\text{rand}}_{1}\times F_{u} & \text{if}\;\;{\text{rand}}_{2}<\tau_{1},\\ F_{i,G} & \text{otherwise}, \end{array}\\ {\text{CR}}_{i,G+1}=\{\begin{array}{cc} {\text{rand}}_{3} & \text{if}\;\;{\text{rand}}_{4}<\tau_{2},\\ {\text{CR}}_{i,G} & \text{otherwise}, \end{array} \end{array}$$

and they produce factors *F* and CR in a new parent vector. rand_*j*_  (*j* ∈ {1,2, 3,4}) are uniform random values within the range [0,1]. *τ*_1_ and *τ*_2_ represent probabilities to adjust factors *F* and CR, respectively. Generally, *τ*_1_ = *τ*_2_ = 0.1, *F*_*l*_ = 0.1, *F*_*u*_ = 0.9. SaDE gave the first attempt to simultaneously adopt more than one mutation scheme in DE [1]. The main propose is to reduce the problem-solving risk by distributing available computational resources to multiple search techniques with different biases. SaNSDE can be regarded as an improved version of the SaDE. Its mutation is executed in the same way as SaDE except that only two mutation schemes are used, and the scale factor *F* in the adopted mutation schemes is generated according to either a Gaussian distribution or a Cauchy distribution [30]. JADE is another recent DE variant, in which a new mutation scheme named “/DE/current-to-pbest” is adopted [31].

## 3. Proposed HPSO-DE

Similar to other EAs, both of PSO and DE are the population-based iterative algorithms. The PSO and DE can easily get trapped in the local optima when solving complex multimodal problems. These weaknesses have restricted wider applications of them. Therefore, avoiding the local optima has become one of the most important and appealing goals in algorithms research. To achieve the goal, an adaptive hybrid algorithm based on PSO and DE (HPSO-DE) for global optimization is proposed. The algorithm focuses on the convergence of population. When a particle discovers a current optima position, the other particles will draw together to the particle. If the position is the local optima, the algorithm will be convergence and clustered in local optima. The premature may appear. Suppose that the population size of HPSO-DE is NP, the fitness value of *i*th particle is *f*_*i*_, and the average fitness value is *f*_avg_. The convergence degree is defined as follows:

(15) $$\begin{array}{c} d=\sqrt{\sum_{i=1}^{NP}{\left(\frac{f_{i}-f_{\text{avg}}}{\max\{1,\max_{1\leq i\leq N}(f_{i}-f_{\text{avg}})}\right)}^{2}.} \end{array}$$

The parameter *d* reflects the convergence degree. When the parameter *d* is large, the algorithm is in random search. On the other hand, the algorithm will get into local optima and the premature maybe occur. In order to evaluate the parameter *d*, *d*_*c*_ is given as follows, where *p* is the mutation probability.

(16) $$\begin{array}{c} p=\{\begin{array}{cc} k, & d<d_{c},\\ 0, & \text{others}. \end{array} \end{array}$$

Generally, *d*_*c*_ ∈ [0.5,2]. If the parameter *d* is less than *d*_*c*_, the mutation probability *p* is equal to *k*. The mutation including PSO and DE is as follows, respectively:

(17) $$\begin{array}{c} g_{\text{best}}=\left(1+0.5\eta \right)g_{\text{best}}, \end{array}$$

(18) $$\begin{array}{c} X_{i,G}=\left(1+0.5\eta \right)X_{i,G}, \end{array}$$

where the parameter *η* obeys Gauss (0,1) distribution.

The strategy makes HPSO-DE enjoy the advantages of two algorithms and maintain diversity of the population. With the efforts, the HPSO-DE has the ability of jumping out of the local optima. The main procedure of HPSO-DE is presented in Algorithm 1.

One of the most important parameters for the proposed HPSO-DE is the balanced parameter *p*. In the following, we will make an integrated analysis of the key parameter by comparing the performance of the HPSO-DE in the optimization of several representative functions.

## 4. Numerical Experiments and Results

### 4.1. Test Functions

16 benchmark functions are used to test the performance of HPSO-DE to assure a fair comparison. If the number of test problems is smaller, it will be very difficult to make a general conclusion. Using a test set which is too small also has the potential risk that the algorithm is biased (optimized) toward the chosen set of problems. Such bias might not be useful for other problems of interest. The benchmark functions are given in Table 1. It denotes the ranges of the variables and the value of the global optimum. Functions *f*_1_ − *f*_16_ are high-dimensional problems. Functions *f*_1_ − *f*_6_ are unimodal. Function *f*_7_ is a noisy quadratic function. Functions *f*_8_ − *f*_16_ are multimodal functions where the number of local minima increases exponentially with the problem dimension [29]. *f*_15_ − *f*_18_ are rotated functions. An orthogonal matrix *M* is generated to rotate a function. The original variable *x* is left-multiplied by the orthogonal matrix *M* to get the new rotated variable *y* = *M* × *x*. This variable *y* is used to compute the fitness value *f*. When one dimension in *x* is changed, all dimensions in *y* will be influenced:

(19) f1=∑i=1Dxi2,f2=∑i=1Dixi2,f3=∑i=1D|xi|+∏i=1D|xi|,f4=∑i=1D(∑j=1ixj)2,f5=max⁡i⁡{|xi|,1≤xi≤D},f6=∑i=1D−1[100(xi+1−xi2)2+(xi−1)2],f7=∑i=1Dixi4+random[0,1),f8=∑i=1D[xi2−10cos⁡(2πxi)+10],f9(x)=∑i=1D(yi2−10cos⁡(2πyi)+10),yi={xi,|xi|<0.5,round(2xi)2,|xi|≥0.5,f10=−20exp⁡(−0.21D∑i=1Dxi2)−exp⁡(1D∑i=1Dcos⁡(2πxi))+20+e,f11=14000∑i=1Dxi2−∏i=1Dcos⁡(xii1/2)+1,f12(x)=πn{10 sin2(πyi)  +∑i=1D(yi−1)2[1+10 sin2(πyi+1)]  +(yn−1)2}+∑i=1Du(xi,10,100,4),yi=(1+14(xi+1)),u(xi,a,k,m)  ={k(xi−a)m,xi>a,0,−a≤xi≤a,k(−xi−a)m,xi<−a,f13(x)=0.1{sin2(3πx1)  +∑i=1D−1(xi−1)2[1+sin2(3πxi+1)]  +(xD−1)2[1+sin2(2πxD)]}+∑i=1Du(xi,10,100,4),f14=∑i=1D(∑k=0kmax⁡[akcos⁡(2πbk(xi+0.5))]   −D∑k=0kmax⁡[akcos⁡(2πbk∗0.5)]),    a=0.5, b=3, kmax⁡=20,f15=∑i=1D[yi2−10cos⁡(2πyi)+10], y=M×x,f16(x)=∑i=1D(zi2−10cos⁡(2πzi)+10),zi={yi,|yi|<0.5,round(2yi)2,|yi|≥0.5,y=M×x,f17=−20exp⁡(−0.21D∑i=1Dyi2)−exp⁡(1D∑i=1Dcos⁡(2πyi))+20+e,  y=M×x,f18=14000∑i=1Dyi2−∏i=1Dcos⁡(yii1/2)+1, y=M×x.

### 4.2. Algorithms for Comparison

Experiments are conducted on a suite of 16 numerical functions to evaluate seven algorithms including the proposed HPSO-DE algorithm. For functions, 30-dimensional (30D) function is tested. The maximum number of function evaluations (FEs) is set to 300 000 and NP is 100. All experiments are run 25 times independently. The seven algorithms in comparison are listed in Table 2.

### 4.3. Comparisons on the Solution Accuracy

**Figure 1:**
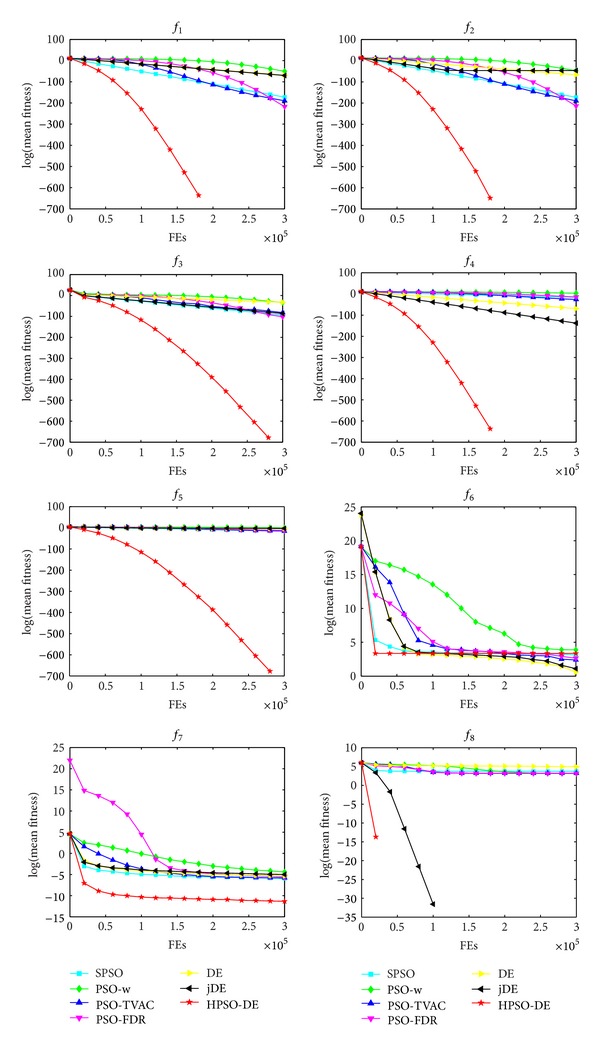
Performance of the algorithms for *f*_1_, *f*_2_, *f*_3_, *f*_4_, *f*_5_, *f*_6_, *f*_7_, and *f*_8_.

**Figure 2:**
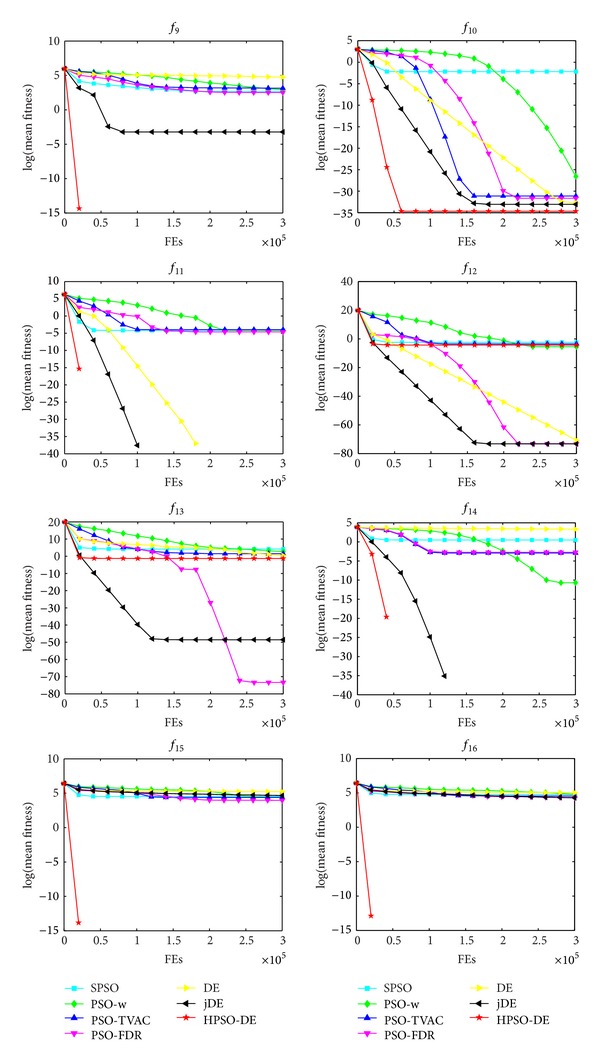
Performance of the algorithms for  *f*_9_, *f*_10_ *f*_11_, *f*_12_, *f*_13_, *f*_14_, *f*_15_, and *f*_16_.

**Figure 3:**
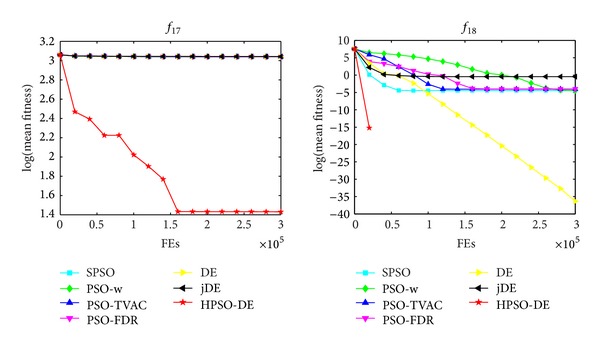
Performance of the algorithms for *f*_17_ and *f*_18_.

The mean and standard deviation (Std) of the solutions in 25 independent runs are listed in Table 3. The best result among these algorithms is indicated by boldface in the table. Figures 1, 2, and 3 show the comparisons in terms of convergence, mean solutions, and evolution processes in solving 16 benchmark functions.

From Table 3 and Figures 1–3, it is very clear that the hybrid proposed algorithm has the strong ability to jump out of the local optima. It can effectively prevent the premature convergence and significantly enhance the convergence rate and accuracy. It provides best performance on the *f*_1_, *f*_2_, *f*_3_, *f*_4_, *f*_5_, *f*_8_, *f*_9_, *f*_11_, *f*_14_, *f*_15_, *f*_16_, and *f*_18_, which reachse the highest accuracy on them. The jDE ranks the second on *f*_8_, and *f*_11_ and performs a little better than HPSO-DE on *f*_6_. PSO-FDR performs best on *f*_12_, *f*_13_.

One can observe that the proposed method can search the optimum and maintain a higher convergence speed. The capabilities of avoiding local optima and finding global optimum of these functions indicate the superiority of HPSO-DE.

### 4.4. Comparisons on Convergent Rate and Successful Percentage

The convergent rate for achieving the global optimum is another key point for testing the algorithm performance. The success of an algorithm means that this algorithm can result in a function value not worse than the prespecified optimal value, that is, for all problems with the number of function evaluations less than the pre specified maximum number. The success rate (SR) is calculated as the number of successful runs divided by the total number of runs.

In Table 4, we summarize the SR of each algorithm and the average number of function evaluations over successful runs (FESS). An experiment is considered as successful if the best solution is found with sufficient accuracy: 10^−8^.

Table 4 shows that HPSO-DE needs least FESS to achieve the acceptable solution on most of functions, which reveals that proposed algorithm has a higher convergent rate than other algorithms. DE and jDE outperform HPSO-DE on the *f*_12_ and *f*_13_; SPSO, LPSO, and PSO-TVAC have much worse SR and accuracy than HPSO-DE on the test functions. In addition, HPSO-DE can achieve accepted value with a good convergence speed and accuracy on most of the functions, as seen from Figures 1–3 and Table 3.

In summary, the HPSO-DE performs best on functions and has good search ability. Owing to the proposed techniques, the HPSO-DE processes capabilities of fast convergence speed, the highest successful rate, and the best search accuracy among these algorithms.

### 4.5. Parameter Study

The balanced parameter *p* needs to be optimized. In this section, we investigate the impact of this parameter on HPSO-DE. The HPSO-DE algorithm runs 25 times on each function with four different balanced parameters of 0, 0.1, 0.2, and 0.3. The influence of balanced parameters on accuracy of HPSO-DE algorithm is investigated by comparing the optima values that HPSO-DE obtains for different balanced parameters.

**Figure 4:**
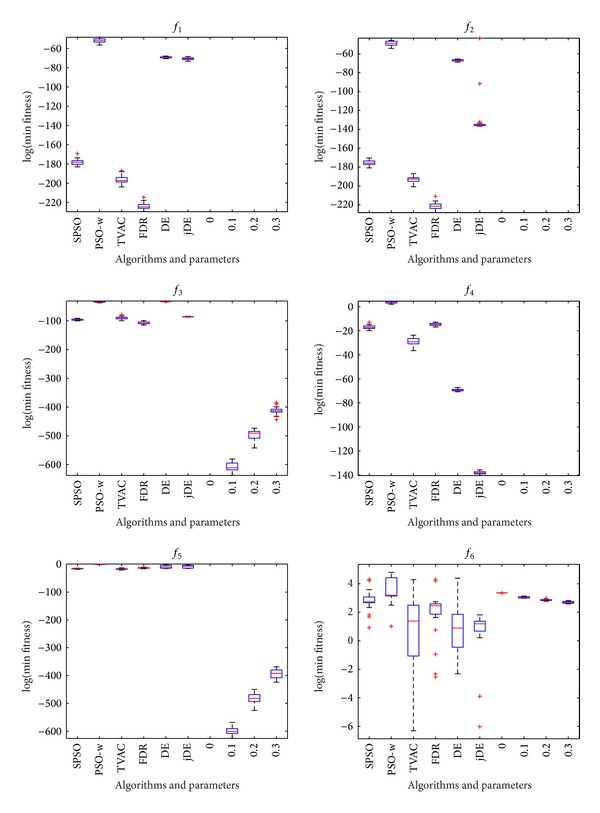
HPSO-DE with different balance parameters and other six algorithms on *f*_1_, *f*_2_, *f*_3_, *f*_4_, *f*_5_, and *f*_6_.

**Figure 5:**
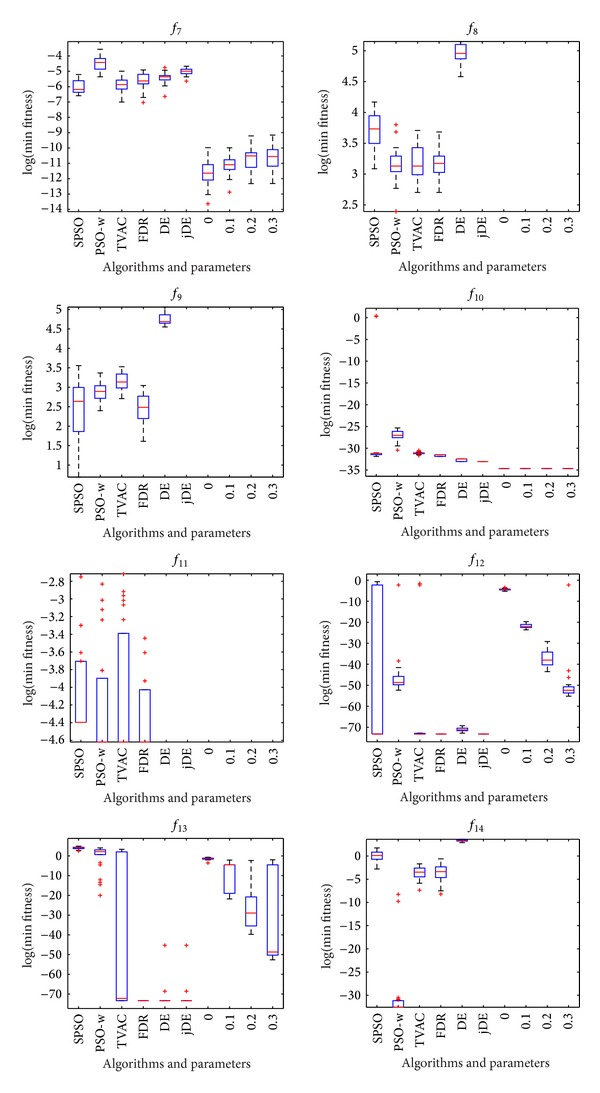
HPSO-DE with different balance parameters and other six algorithms on *f*_7_, *f*_8_, *f*_9_, *f*_10_, *f*_11_, *f*_12_, *f*_13_, and *f*_14_.

**Figure 6:**
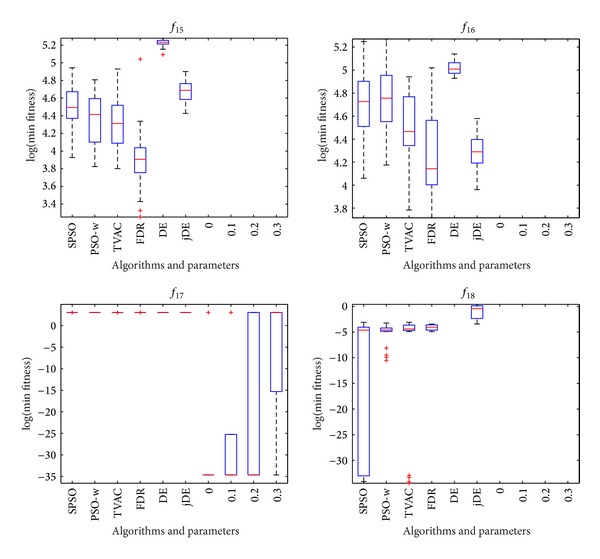
HPSO-DE with different balance parameters and other six algorithms on *f*_15_, *f*_16_, *f*_17_, and *f*_18_.

Figures 4, 5, and 6 show the box plots of minimal values that HPSO-DE obtains with four different balanced parameters. The box has lines at the lower quartile, median, and upper quartile values. The whiskers are lines extending from each end of the box to show the extent of the remaining data. Outliers are data with values beyond the ends of the whiskers.

From Figures 4–6, one can observe that the accuracy of HPSO-DE is less sensitive to the balanced parameter on most of functions except *f*_12_, *f*_13_, and *f*_17_ when balanced parameter is between 0 and 0.3.

### 4.6. Comparison with JADE

The JADE algorithm is tested on a set of standard test functions in [31]. HPSO-DE is compared with JADE on 30D test functions chosen from [31]. The parameter settings are the same as in [31]. Maximum generations are listed in Table 5. The middle results of 50 independent runs are summarized in the table (results for JADE are taken from [31]), which show that the proposed algorithm obviously performs better than the JADE algorithm.

## 5. Conclusions

In this paper, a novel algorithm HPSO-DE is proposed by developing a balanced parameter between PSO and DE. The population is generated either by DE or by PSO according to the balanced parameter. Adaptive mutation is carried out to current population when the population clusters around local optima. The strategy makes HPSO-DE have the advantages of two algorithms and maintain diversity of the population. In comparison with the PSO, DE, and their variants, the proposed algorithm is more effective in obtaining better quality solutions, works in a more effective way, and finds better quality solutions more frequently.

## Figures and Tables

**Algorithm 1:**
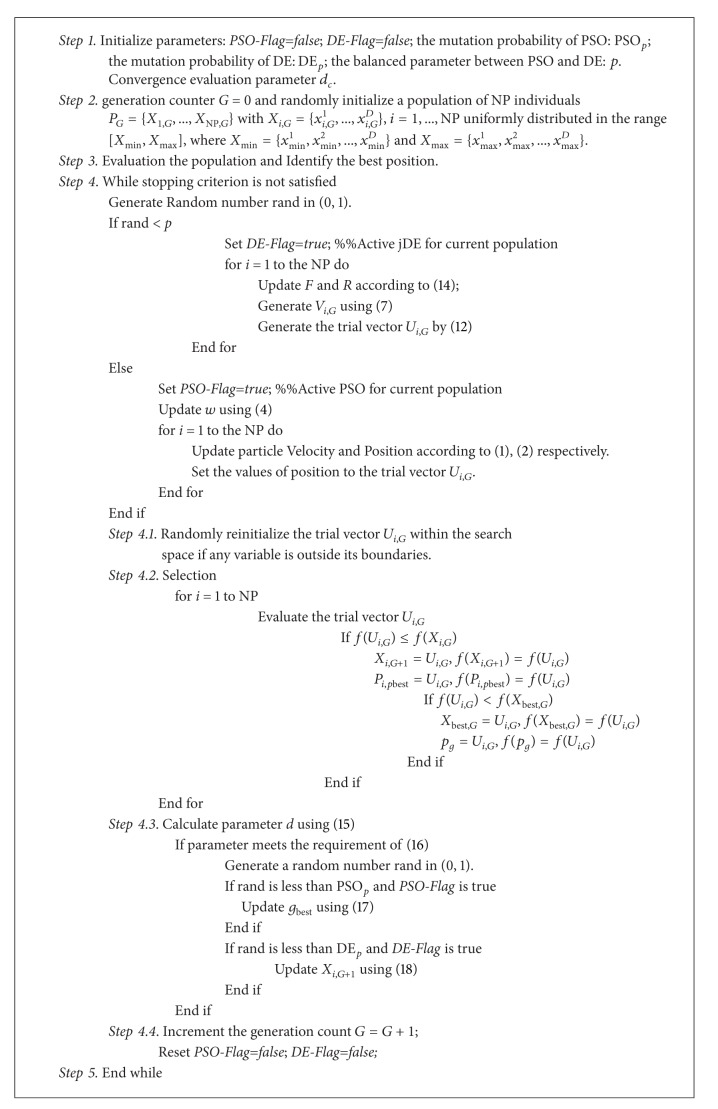


**Table 1:** Benchmark configurations.

Function	Name	Search space	Global optimal *f*(x*)	x*
*f*_1_	Sphere	[−100, 100]	0	0
*f*_2_	Weighted sphere	[−100, 100]	0	0
*f*_3_	Schwefel's Problem 2.22	[−10, 10]	0	0
*f*_4_	Shifted Schwefel's Problem 1.2	[−100, 100]	0	0
*f*_5_	Schwefel's Problem 2.21	[−100, 100]	0	0
*f*_6_	Rosenbrock	[−30, 30]	0	(1, 1,…, 1)
*f*_7_	Quartic	[−1.28, 1.28]	0	0
*f*_8_	Rastrigin	[−5, 5]	0	0
*f*_9_	Noncontinuous Rastigin	[−5, 5]	0	0
*f*_10_	Ackley	[−32, 32]	0	0
*f*_11_	Griewank	[−600, 600]	0	0
*f*_12_	Penalized 1	[−50, 50]	0	0
*f*_13_	Penalized 2	[−50, 50]	0	0
*f*_14_	Weierstrass	[−0.5, 0.5]	0	0
*f*_15_	Rotated Rastrigin	[−5, 5]	0	0
*f*_16_	Rotated noncontinuous Rastrigin	[−5, 5]	0	0
*f*_17_	Rotated Ackley	[−32, 32]	0	0
*f*_18_	Rotated Griewank	[−600, 600]	0	0

**Table 2:** Algorithms initialization.

Algorithm	Parameters	Reference
SPSO	*w* = 0.729, *c*_1_ = *c*_2_ = 1.49	[41]
PSO-w	*w* = 0.9 : 0.4, *c*_1_ = *c*_2_ = 2	[17]
PSO-TVAC	*w* = 0.9 : 0.4, *c*_1_ = 2.5 : 0.5, *c*_2_ = 0.5 : 2.5	[16, 38]
PSO-FDR	*c*_1_ = *c*_2_ = 1, *c*_3_ = 2	[42]
DE	*F* = 0.5, CR = 0.9	[20, 43]
jDE	*F*_*l*_ = 0.1, *F*_*u*_ = 0.9, *τ*_1_ = *τ*_2_ = 0.1	[29]
HPSO-DE	PSO_*p*_ = 0.3, DE_*p*_ = 0.01, *d*_*c*_ = 1.5, *p* = 0	This paper

**Table 3:** Result comparisons among seven algorithms on test functions.

Functions		Algorithms						
		PSO	PSO-w	PSO-FDR	PSO-TVAC	DE	jDE	HPSO-DE
*f*_1_	Mean	1.31*E* − 75	1.61*E* − 22	2.39*E* − 95	4.14*E* − 83	1.04*E* − 30	3.84*E* − 31	0.0**E** + 00
	Std.	4.03*E* − 149	7.47*E* − 44	1.29*E* − 188	1.62*E* − 164	6.81*E* − 61	2.14*E* − 61	0.0**E** + 00

*f*_2_	Mean	7.44*E* − 76	2.80*E* − 21	9.28*E* − 94	4.76*E* − 83	1.33*E* − 29	5.88*E* − 21	0.0**E** + 00
	Std.	4.35*E* − 150	1.47*E* − 41	2.09*E* − 185	2.0*E* − 164	1.36*E* − 58	8.63*E* − 40	0.0**E** + 00

*f*_3_	Mean	2.50*E* − 41	1.99*E* − 15	6.10*E* − 45	3.88*E* − 36	7.51*E* − 15	5.57*E* − 038	0.0**E** + 00
	Std.	3.36*E* − 81	1.15*E* − 29	6.64*E* − 88	3.68*E* − 70	5.05*E* − 29	1.45*E* − 75	0.0**E** + 00

*f*_4_	Mean	1.83*E* − 7	4.70*E* + 1	6.77*E* − 7	5.74*E* − 12	1.17*E* − 30	1.80*E* − 60	0.0**E** + 00
	Std.	2.21*E* − 13	9.39*E* + 2	5.42*E* − 13	1.80*E* − 22	1.92*E* − 60	6.01*E* − 120	0.0**E** + 00

*f*_5_	Mean	2.63*E* − 7	1.0*E* + 0	4.26*E* − 6	1.91*E* − 7	1.59*E* − 2	5.92*E* − 2	0.0**E** + 00
	Std.	5.79*E* − 14	3.0*E* − 1	5.12*E* − 11	2.52*E* − 13	8.59*E* − 4	3.31*E* − 2	0.0**E** + 00

*f*_6_	Mean	2.24*E* + 1	4.89*E* + 1	1.41*E* + 1	1.10*E* + 1	1.10*E* + 1	3.04**E** + 0	2.86*E* + 2
	Std.	3.69*E* + 2	1.53*E* + 3	3.14*E* + 2	3.50*E* + 2	5.09*E* + 2	2.39*E* + 0	9.0**E** − 3

*f*_7_	Mean	2.0*E* − 3	1.2*E* − 2	4.0*E* − 3	3.0*E* − 3	4.0*E* − 3	7.0*E* − 3	1.2**E** − 5
	Std.	1.16*E* − 6	2.60*E* − 5	3.18*E* − 6	2.01*E* − 6	2.33*E* − 6	1.70*E* + 0	1.049**E** − 10

*f*_8_	Mean	4.31*E* + 1	2.44*E* + 1	2.45*E* + 1	2.57*E* + 1	1.42*E* + 2	0.0**E** + 00	0.0**E** + 00
	Std.	1.31*E* + 2	4.94*E* + 1	3.27*E* + 1	6.10*E* + 1	5.11*E* + 2	0.0**E** + 00	0.0**E** + 00

*f*_9_	Mean	1.37*E* + 1	1.86*E* + 1	1.24*E* + 1	2.33*E* + 1	1.16*E* + 2	4.0*E* − 2	0.0**E** + 00
	Std.	7.75*E* + 1	1.90*E* + 1	1.61*E* + 1	2.43*E* + 1	2.82*E* + 2	4.0*E* − 2	0.0**E** + 00

*f*_10_	Mean	1.14*E* − 1	2.92 − *E*12	1.82*E* − 14	3.08*E* − 14	6.29*E* − 15	4.44*E* − 15	8.882**E** − 16
	Std.	1.55*E* − 1	6.88*E* − 24	1.30*E* − 29	8.60*E* − 29	3.28*E* − 30	0.0**E** + 00	0.0**E** + 00

*f*_11_	Mean	1.5*E* − 2	1.5*E* − 2	1.0*E* − 2	1.9*E* − 2	0.0**E** + 00	0.0**E** + 00	0.0**E** + 00
	Std.	2.44*E* − 4	3.0*E* − 4	8.97*E* − 5	4.40*E* − 4	0.0**E** + 00	0.0**E** + 00	0.0**E** + 00

*f*_12_	Mean	9.1*E* − 2	4.0*E* − 3	1.57**E** − 32	2.90*E* − 2	1.87*E* − 31	1.58*E* − 32	1.3*E* − 2
	Std.	3.2*E* − 2	4.0*E* − 4	3.12**E** − 95	4.0*E* − 3	3.39*E* − 62	3.12**E** − 95	3.30*E* − 5

*f*_13_	Mean	6.34*E* + 1	1.45*E* + 1	1.35**E** − 32	4.44*E* + 0	1.49*E* + 0	8.33*E* − 22	2.76*E* − 1
	Std.	1.07*E* + 3	2.13*E* + 2	3.12**E** − 95	5.26*E* + 1	1.68*E* + 1	1.73*E* − 41	1.2*E* − 2

*f*_14_	Mean	1.67*E* + 0	2.28*E* − 5	6.65*E* − 2	5.5*E* − 2	2.79*E* + 1	0.0**E** + 00	0.0**E** + 00
	Std.	2.03*E* + 0	5.10*E* − 9	1.18*E* − 2	3.0*E* − 3	2.78*E* + 1	0.0**E** + 00	0.0**E** + 00

*f*_15_	Mean	9.25*E* + 1	8.24*E* + 1	5.23*E* + 1	8.01*E* + 1	1.86*E* + 2	1.07*E* + 2	0.0**E** + 00
	Std.	4.84*E* + 2	5.01*E* + 2	5.99*E* + 2	6.77*E* + 2	5.82*E* + 1	1.97*E* + 2	0.0**E** + 00

*f*_16_	Mean	1.17*E* + 2	1.20*E* + 2	7.52*E* + 1	9.38*E* + 1	1.52*E* + 2	7.29*E* + 1	0.0**E** + 00
	Std.	1.33*E* + 3	1.00*E* + 3	8.81*E* + 2	7.02*E* + 2	9.76*E* + 1	1.28*E* + 2	0.0**E** + 00

*f*_17_	Mean	2.09*E* + 1	2.09*E* + 1	2.09*E* + 1	2.09*E* + 1	2.09*E* + 1	2.10*E* + 1	4.18**E** + 0
	Std.	2.6*E* − 3	4.3*E* − 3	2.2*E* − 3	2.1*E* − 3	1.80**E** − 3	2.2*E* − 3	7.29*E* + 1

*f*_18_	Mean	1.15*E* − 2	1.17*E* − 2	1.87*E* − 2	1.76*E* − 2	1.60*E* − 16	6.47*E* − 1	0.0**E** + 00
	Std.	1*E* − 4	1*E* − 4	1*E* − 4	2*E* − 4	2.06*E* − 31	2.74*E* − 1	0.0**E** + 00

**Table 4:** Convergence speed and algorithm reliability comparisons.

Functions		Algorithms						
		PSO	PSO-w	PSO-FDR	PSO-TVAC	DE	jDE	HPSO-DE
*f*_1_	FESS	4.5056*E* + 4	2.3642 + *E*5	1.4738*E* + 5	1.0142*E* + 5	1.1055*E* + 5	1.0883*E* + 5	2.2075**E** + 4
	SR	100%	100%	100%	100%	100%	100%	100%

*f*_2_	FESS	4.9428*E* + 4	2.4217*E* + 5	1.5194*E* + 5	1.0435*E* + 5	1.1905*E* + 5	6.4498*E* + 4	2.3002**E** + 4
	SR	100%	100%	100%	100%	100%	100%	100%

*f*_3_	FESS	6.8336*E* + 4	2.5089*E* + 5	1.6382*E* + 5	1.1524*E* + 5	1.8979*E* + 5	7.6644*E* + 4	3.2762**E** + 4
	SR	100%	100%	100%	100%	100%	100%	100%

*f*_4_	FESS	2.907*E* + 5	—	—	2.4757*E* + 5	1.1041*E* + 5	5.9383*E* + 4	2.2075**E** + 4
	SR	12%	0%	0%	**100%**	**100%**	**100%**	100%

*f*_5_	FESS	2.987*E* + 5	—	—	2.8725*E* + 5	—	—	3.2629**E** + 4
	SR	4%	0%	0%	24%	0%	0%	100%

*f*_8_	FESS	—	—	—	—	—	7.3477*E* + 4	2.3084**E** + 4
	SR	0%	0%	0%	0%	0%	100%	100%

*f*_9_	FESS	—		—	—	—	8.7133*E* + 4	2.2356**E** + 4
	SR	0%		0%	0%	0%	96%	100%

*f*_10_	FESS	7.337*E* + 4	2.7072*E* + 5	1.7226*E* + 5	1.2118*E* + 5	1.7113*E* + 5	9.0429*E* + 4	3.2502**E** + 4
	SR	92%	**100%**	**100%**	**100%**	**100%**	**100%**	100%

*f*_11_	FESS	4.6325*E* + 4	2.3907*E* + 5	1.5296*E* + 5	1.0411*E* + 5	1.1398*E* + 5	6.2468*E* + 4	2.0659*E* + 4
	SR	32%	28%	36%	36%	**100%**	**100%**	**100%**

*f*_12_	FESS	4.6978*E* + 4	2.4440*E* + 5	1.3834*E* + 5	1.0872*E* + 5	1.0199*E* + 5	5.0201**E** + 4	—
	SR	72%	96%	**100%**	80%	**100%**	**100%**	0%

*f*_13_	FESS	—	2.975*E* + 5	1.6684*E* + 5	1.4981*E* + 5	2.5882*E* + 5	5.7271**E** + 4	—
	SR	0%	4%	**100%**	60%	52%	**100%**	0%

*f*_14_	FESS	—	2.7346*E* + 5	—	—	—	8.435*E* + 4	** 3.85E+4**
	SR	0%	88%	0%	0%	0%	**100%**	100%

*f*_15_	FESS	—	—	—	—	—	—	2.1025**E** + 4
	SR	0%	0%	0%	0%	0%	0%	100%

*f*_16_	FESS	—	—	—	—	—	—	2.2563**E** + 4
	SR	0%	0%	0%	0%	0%	0%	100%

*f*_17_	FESS	—	—	—	—	—	—	6.8878**E** + 4
	SR	0%	0%	0%	0%	0%	0%	80%

*f*_18_	FESS	1.0931*E* + 5	—	—	1.4448*E* + 5	1.834*E* + 5	—	2.268**E** + 4
	SR	36%	0%	0%	20%	100%	0%	**100%**

**Table 5:** Results of the comparison between HPSO-DE and JADE.

Functions	Gen	HPSO-DE Mean (Std Dev.)	JADE w/o archive Mean (Std Dev.)	JADE with archive Mean (Std Dev.)
*f*_1_	1500	2.9**E** − 121** **(1.0**E** − 240)	1.8*E* − 60 (8.4*E* − 60)	1.3*E* − 54 (9.2*E* − 54)
*f*_3_	2000	1.7**E** − 78** **(4.5**E** − 155)	1.8*E* − 25 (8.8*E* − 25)	3.9*E* − 22 (2.7*E* − 21)
*f*_4_	5000	0.0**E** + 00** **(0.0**E** + 00)	5.7*E* − 61 (2.7*E* − 60)	6.0*E* − 87 (1.9*E* − 86)
*f*_5_	5000	1.7**E** − 185** **(00.0**E** + 00)	8.2*E* − 24 (4.0*E* − 23)	4.3*E* − 66 (1.2*E* − 65)
*f*_6_	3000	2.9 + *E*2 (1.1*E* − 2)	8.0**E** − 02** **(5.6**E** − 01)	3.2*E* − 01 (1.1*E* + 00)
	20000	2.9 + *E*2 (3.4*E* − 2)	8.0**E** − 02** **(5.6**E** − 01)	3.2*E* − 01 (1.1*E* + 00)
*f*_7_	3000	2.1**E** − 5** **(2.2**E** − 10)	6.4*E* − 04 (2.5*E* − 04)	6.8*E* − 04 (2.5*E* − 04)
*f*_8_	1000	0.0**E** + 00** **(0.0**E** + 00)	1.0*E* − 04 (6.0*E* − 05)	1.4*E* − 04 (6.5*E* − 05)
	5000	0.0**E** + 00** **(0.0**E** + 00)	0.0**E** + 00** **(0.0**E** + 00)	0.0**E** + 00** **(0.0**E** + 00)
*f*_10_	500	8.9**E** − 16** (**0.0**E** + 00)	8.2*E* − 10 (6.9*E* − 10)	3.0*E* − 09 (2.2*E* − 09)
	2000	8.9**E** − 16** **(0.0**E** + 00)	4.4*E* − 15 (0.0*E* + 00)	4.4*E* − 15 (0.0*E* + 00)
*f*_11_	500	0.0**E** + 00** **(0.0**E** + 00)	9.9*E* − 08 (6.0*E* − 07)	2.0*E* − 04 (1.4*E* − 03)
	3000	0.0**E** + 00** **(0.0**E** + 00)	0.0**E** + 00** **(0.0**E** + 00)	2.0*E* − 04 (1.4*E* − 03)
*f*_12_	500	1.8*E* − 2 (1*E* − 4)	4.6**E** − 17** **(1.9**E** − 16)	3.8*E* − 16 (8.3*E* − 16)
	1500	1.7*E* − 2 (4*E* − 4)	1.6**E** − 32** **(5.5**E** − 48)	1.6**E** − 32** **(5.5**E** − 48)
*f*_13_	500	4.4*E* − 1 (2.1*E* − 2)	2.0**E** − 16** **(6.5**E** − 16)	1.2*E* − 15 (2.8*E* − 15)
	1500	3.1*E* − 1 (1.6*E* − 2)	1.4**E** − 32** **(1.1**E** − 47)	1.4**E** − 32** **(1.1**E** − 47)
